# Switchable 3D optofluidic Y-branch waveguides tuned by Dean flows

**DOI:** 10.1038/srep38338

**Published:** 2016-12-02

**Authors:** L. Li, X.Q. Zhu, L. Liang, Y. F. Zuo, Y. S. Xu, Y. Yang, Y. J. Yuan, Q. Q. Huang

**Affiliations:** 1 School of Physics & Technology, Wuhan University, Wuhan 430072, China; 2 School of pharmaceutical sciences, Wuhan University, Wuhan 430072, China

## Abstract

Optical branch waveguides are one of the most important optical elements and have been widely exploited for optical communication systems. However, prevailing devices are typically solid and have limit in tunability. Liquid optical devices have attracted more interest for the advantage of tunability of liquid media, but their signals suffer serious leakage if the refractive index (RI) of liquid is smaller than that of solid channels. This paper demonstrates the tunable three-dimensional (3D) optofluidic Y-branch waveguides in plannar microchannels by simply introducing Dean flow. This device can reconfigure 3D Y-branch profiles and separate the intensity of light as tunable ratio from 0 to 1 by adjusting the flow rates with low loss. Different from the prevailing 2D liquid counterparts, the 3D configuration offer much more freedom in the selection of liquid media as liquid’s RI can be totally independent to the solid channel structure. The transmission loss through the device is estimated to 0.97 db when the splitting angle is 10°, which shows the light is confined better in the 3D liquid structures than traditional 2D liquid counterparts. The Y-branch waveguides show potential in applications of integrated optofluidic devices.

Branch waveguides play an important role in optical integrated systems and have been widely applied in many ways such as optical switches, Mach–Zehnder interferometer and power splitters, etc^1^,^2^. Different from traditional single waveguides, typically branch waveguides usually consist of a stem waveguide and two diverging arm waveguides. Light can be separated when light travel through two arms. It is one of the cheapest and simplest methods to divide light power into two portions. However, the branch waveguides have great limit tunability of refractive index (RI) and shape because of the use of solid materials^3^. Some solutions have been raised such as acousto-optic branch waveguides^4^, magneto-optic branch waveguides^5^, electro-optic branch waveguides^6^, thermo-optic effect^7^,^8^ and micro-electro-mechanical systems (MEMS) technology^9^,^10^ to overcome this disadvantage. But the fabrication is complex and there is still difficulty in large range tunability.

Optofluidics work on the synergy between optics and microfluidics, many innovative optical devices have been created^11^, such as liquid waveguide^12^,^13^,^14^, liquid lens & prism^15^,^16^, flow cytometers^17^,^18^, liquid switches^19^,^20^,^21^, dye laser^22^,^23^, and self-imaging^24^, *et al*. In these devices, liquid media can be easily replaced and reconfigured, exhibiting pretty larger tunability than solid ones. However, prevailing liquid optical ones in planar microchannels are typically two-dimensional (2D), their optical signals will suffer serious leakage if the RI of liquid is smaller than that of solid channel wall. In other words, traditional optofluidic devices cannot be independent as the RI of core liquid must be greater than that of channel to avoid the light escaping into solid substrate^12^,^13^. This is an inherent drawback for communication systems, especially for branch waveguides^14^,^25^.

Dean flows are usually used in chemical mixing or particles separation^26^,^27^. Inside a curvilinear channel, fluids experience centrifugal effects exerted along its radial direction and inertial forces resulted from its axial motion which can form a radial pressure gradient. The transverse flows can be generated by the interplay of these two effects. It can reconfigure microfluidics in three-dimensional (3D) profile^28^,^29^. Recently, single 3D waveguides have been fabricated in microchannel by centrifugal effect^22^,^30^, but more complex elements such as branch waveguides are desired for all optics circuit.

In this paper, we demonstrate a pure liquid 3D branch waveguide via Dean flows. Unlike the traditional solid counterparts, the tunable 3D profiles are achieved via transverse Dean flows. The device can realize tunable optical switching and dividing properties through adjusting the flow rates of two liquid with different RI. Besides, different from the 2D liquid ones, the 3D configuration offers much more freedom in the selection of liquid media as RI of liquids can be independent of the solid channel. At the same time, the 3D branch waveguide still have the common advantages like the high degree of integration and small consumption. It shows that the branch waveguide could have wider applications such as the optical attenuator and 1 × 2 optical switch^31^ in integrated optical systems.

## Methods

### Design and theoretical analysis

**Figure 1:**
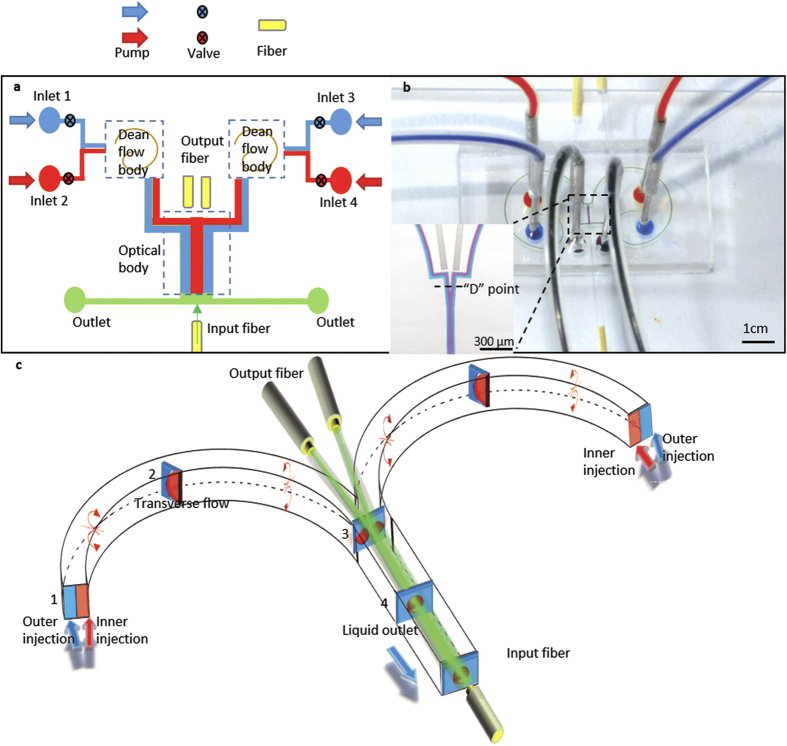
The schematic of the 3D optofluidic Y-branch waveguides tuned by Dean flows. (**a**) System of this 3D optofluidic Y-branch waveguides. (**b**) A picture of the microchip. Food dyes were added to show the inlets, outlets and channels. The mini picture is the local magnified image of the dotted box in the big picture. (**c**) A stereo schematic of the device. Through the curving microchannel, the light can propagate in it and be separated into two branches.

Figure 1a and b show the system of this 3D optofluidic Y-branch waveguides. It has four inlets dyed with blue and red to inject liquid by external pumps. Two Dean flow bodies composed of different circular arc produce centrifugal effect. The optical body can direct the light from the input fiber to output fibers. The color of fibers is yellow. And there are two outlets to effluent discharge. The “D” point is the position to calculate the Dean number. Figure 1c shows the schematic of the Dean flow-based tunable Y-branch waveguides. The design is consists of two symmetric curved and one Y-shape junction microchannel. Four streams flowing side by side are injected into the microchannel (inset 1). The two flowing streams (Red & blue) are influenced by the Dean flow with the counter-rotating liquid cross section in the curving microchannel (inset 2). The inner wall flowing stream may be transported to the outer wall by the transverse flow but the outer one is pushed inward. As a consequence, the relative locations of both flowing streams are changed. In some cases, the flow of the internal flow is completely surrounded by the outer liquid flow (inset 3). Two single 3D optofluidic liquid core-liquid cladding waveguides can be formed if the inner flowing streams’ RI is higher than the outer flowing streams. The two 3D liquid core-liquid cladding waveguides will combine in the Y-shape junction, and in the following straight microchannel the structure of the liquid is maintained downstream (inset 4). As a result, a 3D optofluidic Y-branch waveguide is completed by tuning the Dean flows. The incident light will propagate in the 3D Y-branch even the RI of the liquid are less than that of solid channel. Besides, switching between the “on” state and the “off” state is a necessity for the optical communication devices. Nevertheless, almost all the Y-branches demonstrated so far are stationary rather than real-time switchable. In this device, changing the flow rate of the injected liquids can flexibly tune the structure of liquid. When the flow rates are low, the 3D Y-branch waveguides will vanish, the light will be leaked to solid channel without propagating and splitting in the Y-junction. Besides, changing the flow rate of different branches will influence the volume ratio of the Y-branches, the splitting ratio also can be tuned.

Dean number (De) is a dimensionless number can be used to character the Dean flow. It means the trend of the Dean flow. The bigger the De is, the larger tendency of relative motion the transverse flow has. There are two dimensionless parameters that govern the Dean number, the Reynolds number and the pipe-to-curvature radius ratio. The Reynolds number is defined as


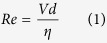


in which the 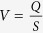 is the average axial velocity of the flow streams (Q is the flow rate and S is the cross section area of the microchannel), η is an average kinematic viscosity of two flow streams and d is the width of the microchannel in our device. The pipe-to-curvature radius ratio is expressed as


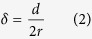


where the r is a geometrical parameter referring to the channel radius of curvature. Dean number (De) is defined as^32^.


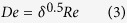


when the liquids are fixed, the Dean number is determined by both the geometrical construction of the curved channel and the flow rates of the injected liquids. Larger centrifugal effects will be leaded by the higher Dean number and the action time for the Dean flow is relatively longer due to the long flow path.

The numerical aperture (NA) is usually used to describe the capability of collecting light for the circular optical waveguide. Bigger NA means stronger collecting light capability. It is a dimensionless parameter defined as
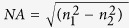
, n_1_ is the RI of the core of the waveguide and n_2_ is the RI of the cladding of the waveguide (n_1_ is generally lager than n_2_). The NA of input fiber we used in the chip is 0.12 and the core diameter of the fiber is 9 μm. The NA and the core diameter of the optofluidic branch waveguide should not less than the input fiber for collecting light. So the selection of the liquid media and the size of the branch waveguide must with a view to above parameters when we design the micro device. The property of modes and radial distribution of field in an optical waveguide has direction correlation with the RI distribution and the wavelength of the light and can be simplified analyzed by the normalized frequency. The normalized frequency (V) is defined as^33^


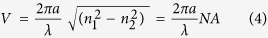


or can be expressed as


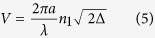


where the *a* is the transverse feature size of the optical waveguide, the *λ* is the wavelength of the input laser and the 
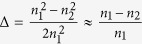
, means the relative RI difference between the cladding and the core. In the 3D branch waveguide, the *a* can be regard as the radius of the core. The larger *V*, the more guide modes can be propagated into the optical waveguide. Taking the circular fibers for the example, when the *V* is no more than 2.4048, a fiber can be seemed as a single mode fiber, otherwise there will be more than one mode propagating in a fiber. Benefited from the greater rangeability in shape and RI compared to the solid media, the *NA* and *V* are also can be adjusted in the 3D optofluidic branch waveguide. It means the tunability of the branch waveguide will be excellent.

### Materials and fabrication

The current soft lithography process is used to fabricate the tunable optofluidic Y-branch waveguides chip. Step one is spin-coating a 150-μm photoresist layer (Micro-Chem, SU8-2050) onto a silicon wafer, the room temperature is about 22 °C, the spin speed is 1000 rpm/min and the spin time is one minute. Step two is the pre-baking on a level hotplate at 65 °C for 5 minutes and 95 °C for 25 minutes. Step three is the exposure, the sample is exposed to recommend UV light by the mask aligner (OAI, 506) under a glass mask. Step four is post exposure bake (65 °C for 5 minutes and 95 °C for 10 minutes). Step five, developing the sample for 15 minutes. Step six is hard bake (150 °C for 10 minutes). After the hark bake, the SU8 mode is fabricated. Then, microchannels are molded using polydimethylsiloxane (PDMS, Momentive RTV615) and bonded to a flat PDMS sheet using plasma cleaner (Harrick, PDC-002). At last an oven is used to bake the bonded PDMS chip at 75 °C for at least one hour to strengthening the adhesion. PDMS is excellent optical transparence material for optofluidic chip.

In the experiment, four flows are injected into the microchannel by the syringe pumps (Longer Pump, LSP01-2A) from different inlets. *Q*_*i*_ (*i* = *1, 2, 3, and 4*) are used to represent the flow rate of each inlet in Fig. 1a. The splitting angle of the Y-junction is 10°. The inner injected liquid is chosen as Ethylene glycol (EG) solution and outer injected liquid is deionized (DI) water. The RI of the inner flowing streams is *n*_*1*_ = *1.3605* (28% (CH_2_OH)_2_ and 72% (H_2_O) in mass, *η*_*1*_ = *2.12 mm*^*2*^*•s*). The RI of outer flowing stream is *n*_*2*_ = *1.332* (DI water, *η*_*2*_ = *1 mm*^*2*^*•s*). Both of them are smaller than that of PDMS channel. The refractometer (ATAGO, PAL-RI) and the viscometer (YOKE, NDJ-8S) are used to measure the RI and viscosity of the liquid, respectively. The operation wavelength of the refractometer is 589 nm and the room temperature is about 20 °C. The RI fluctuation of the two liquid in the range of visible light is negligible so the refractometer is widely used to measure the liquid RI in visible wavelengths.

## Results

**Figure 2:**
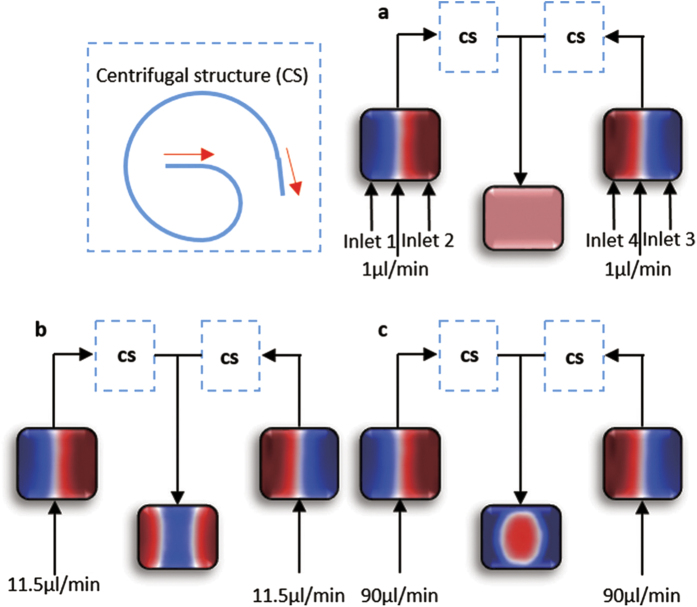
The comparison simulation flow chart of the inlets and the junction with different flow rates. Two colors display different liquid with different RI. They are all injected from the four inlets and are eliminated from the outlet. The centrifugal structure in the dotted box can produce centrifugal effect. Every picture is composed of three small pictures. The upper two are the flow profile of the inlets. The under one is the flow profile in the junction of the device. The flow rates are (**a**) *Q*_*1*_ = *Q*_*2*_ = *Q*_*3*_ = *Q*_*4*_ = *1ul/min*, (**b**) *Q*_*1*_ = *Q*_*2*_ = *Q*_*3*_ = *Q*_*4*_ = *12.5* *ul/min* and (**c**) *Q*_*1*_ = *Q*_*2*_ = *Q*_*3*_ = *Q*_*4*_ = *95 ul/min*. The different flows in (**a**) are mixed. The diffusion in (**b**) is slight but the structure of liquid is still a 2D waveguide. In 2c the diffusion can be ignored and the high RI liquid (red) is surrounded by the low RI liquid (blue) and form the 3D liquid branch waveguides.

The shape of the fluidic interface can be altered by changing the flow rates. Higher flow rates generate a higher Dean number, causing stronger interface reconfiguration. Figure 2a–c show the simulated results with different flow rates. The finite element method is introduced in our simulations to build the model. The high RI liquid is signed with red and the low RI liquid is signed with blue. The centrifugal structure is displayed in the dotted box. Two arcs with different radius are taken together. The center line radius of the smaller arc is 1500 μm and the radian of the smaller arc is 180°. The center line radius of the bigger arc is 2500 μm and the radian of the smaller arc is 265°. And the length of the junction is set as 1100 μm. For the most part, the width and height of the channel is 100 μm. But in the junction of the device, the width is set as 150 μm. The wall of the channel is set as non-slipping and the liquid is incompreible. The true microchip is basically the same with the simulation model except a right-angled channel for the output fiber. As showed in Fig. 2a, when the flow rates are so low, *Q*_*1*_ = *Q*_*2*_ = *Q*_*3*_ = *Q*_*4*_ = *1 μl/min*, that the centrifugal effect can be neglected and the De approaches 0. In this state, the diffusion is dominant and the two liquid mixes completely before they reach the Y-junction. In Fig. 2b, when the flow rate increases to 12.5 μl/min, the position of inner channel is still occupied by the EG flow stream, and the position of outer channel is the DI water. It can be explained by the fact that diffusion effect is tiny and the centrifugal effect is also too tiny to change the liquid position. With the flow rate reaching to *Q*_*1*_ = *Q*_*2*_ = *Q*_*3*_ = *Q*_*4*_ = *95 μl/min* as it shows in Fig. 2c, the centrifugal effect is obvious and the 3D Y-branch profiles are fabricated.

In order to obtain the 3D profile reconfiguration of the liquid after the curving microchannel, a laser scanning confocal microscopy (Nikon, A1R) is conducted to observe the liquid profiles in the Y-junction microchannel. The rhodamineB is dissolved in EG solutions with a concentration of 0.6 mM while rhodamine 6 G is dissolved in DI water with a concentration of 0.6 mM. DPSS and argon ion laser (the operation wavelength respectively are 561 nm, 488 nm) are inserted for fluorescence excitation. The inner flow stream dyed with rhodamine B and the outer flow stream dyed with rhodamine 6 G respectively looks like red and blue in the fluorescent images. The 3D structure of the four flow streams is scanned using a Z-stacked mode at 3-μm interval with an inverted optical microscope and a 10 × /0.30 NA objective lens (Nikon).

**Figure 3:**
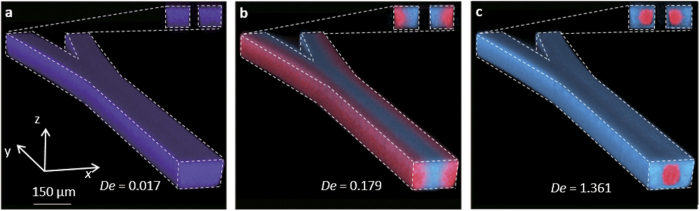
The images of confocal microscopy in different flow rates. The main part is around the Y-shape junction and the mini picture shows the cross section of the junction. The flow rates are (**a**) *Q*_*1*_ = *Q*_*2*_ = *Q*_*3*_ = *Q*_*4*_ = *1* *μl/min*, (**b**) *Q*_*1*_ = *Q*_*2*_ = *Q*_*3*_ = *Q*_*4*_ = *12.5* *μl/min*, (**c**) *Q*_*1*_ = *Q*_*2*_ = *Q*_*3*_ = *Q*_*4*_ = *95 μl/min*.

Figure 3 shows the 3D fluidic profiles of the Y-junction, which illustrates the reconfiguration processes at D point, when the Dean numberis increased from 0.017 to 1.361. Because there is no centrifugal effect in the straight channel, the structure of the liquid can be maintained when they pass the junction. As a result, the De number at point “D” can be used to measure the centrifugal effect of the curved channels. The flow rates of Fig. 3a–c are same to Fig. 2a–c, respectively. When the Dean number is very low (*De* = *0.017*), diffusion is intense and the two flow streams mixed together, giving out purple fluorescent signal as it shown in Fig. 3a. In Fig. 3b, where the flow rates are increased to 12.5 μl/min (*De* = *0.179*) the interfaces between different flow streams are clear. In Fig. 3c, it can be clearly seen that the position of the liquid with high RI are changed from the side to the core when the flow rate are increased to 95 μl/min (*De* = *1.361*) and the diffusion is weak that can be ignored. Finally the EG stream are surrounded by the DI water steams and then they can form the 3D Y-branches. In addition, if the flow rates are further increased, liquid will be excessively swung, so the structure of the 3D liquid branches will be broken. Table 1 summarizes six typical 3D profiles to recurrence the change process of Dean flows from 1 μl/min (*De* = *0.017*) to 135 μl/min (*De* = *1.933*). The cross section is located at the end of straight part. The right picture is the experiment result and the left is the simulation. From these results, the traces of the transverse flow can be tracked, and they can construct more complex 3D profiles via different Dean Numbers. It implies that more 3D optical elements may achieve in future.

**Figure 4:**
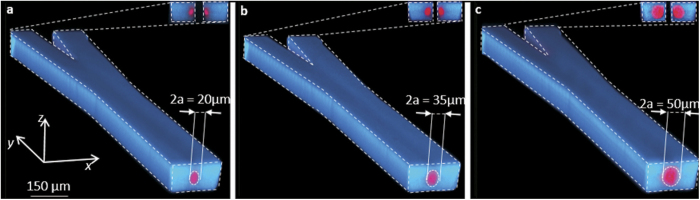
The images of confocal microscopy in different volume ratio. The main part is around the Y-shape junction and the mini picture shows the cross section of the junction. The totally flow rate is constant. 2a means the diameter of the core flow. The ratios of core flow rate to the cladding flow rate are (**a**) 1:4, (**b**) 1:2 and (**c**) 1:1.

Due to the reconfigurability of the liquid, the structure of the 3D optofluidic branch waveguide by dean flows also has excellent tunability. Figure 4a–c show the size of the core of the branch waveguide can be changed by adjusting the volume ratio of the injected liquid. In Fig. 4a, the flow rate of the core liquid is 38 μl/min and the flow rate of the cladding liquid is 152 μl/min. The volume ration between them is 1:4 and the diameter of the core is about 20 μm. When the volume ratio is increased from 1:4 (shown in Fig. 4a) to 1:1 (shown in Fig. 4c), the liquid core is enlarged and its diameter is increased from 20 μm to 50 μm. If the RI of the liquid and the wavelength of the input laser are constant, the variation of the core diameter will change the normalized frequency of the branch waveguide according to the equation (4). In our experiments, n_1_ and n_2_ have been given above as *n*_*1*_ = *1.3605, n*_*2*_ = *1.332 (Δ* = *0.021*) and the λ of the input laser is 532 nm. For the diameter change from 20 μm to 50 μm, the V is increased from 32.7 to 81.8. So the entrance of the 3D optofluidic branch waveguide can be seemed as a multimode waveguide. But if the λ stay the same and the 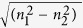 is lower than 0.0203 (for *a* = *10 μm) or* the 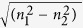 is lower than 0.00814 (for *a* = *25 μm)*, the V will be lower than 2.4048 so the 3D optofluidic branch waveguide can be seemed as a single mode waveguide. According to the equation (4) and taking the V at the critical value *V* = *2.4048*, generates a new equation about the *λ*_*c*_^33^.


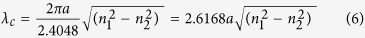


where *λ*_*c*_ is the cutoff wavelength. For a single mode fiber, the operating wavelength must be bigger than the *λ*_*c*_. For a multimode fiber, its operating wavelength is usually less than the *λ*_*c*_. When the radius of the core liquid is *a* = *10–25 μm,* the cutoff wavelength can be calculate as *λ*_*c*_ = *7.249–18.121 μm.* So if the λ of incident laser is larger than the *λ*_*c*_, the 3D optofluidic branch waveguide can also be seemed as a single mode waveguide. It means the collecting light capability of the 3D optofluidic branch waveguide can be easily adjusted thanks to the liquid material. In our experiments, the deformation of the PDMS is unconspicuous (Re < 30) by comparing the confocal photomicrograph from low flow rate to high flow rate. Although there may be tiny perturbation we think it should be acceptable in the range of permitted errors. In addition, the confocal images requires the distribution of the liquid maintaining stable. In the process of an experiment, there are dozens of images need to be captured and each one cost several minutes. It demonstrates the stability of this optofluidic system is good enough to realize its function.

**Figure 5:**
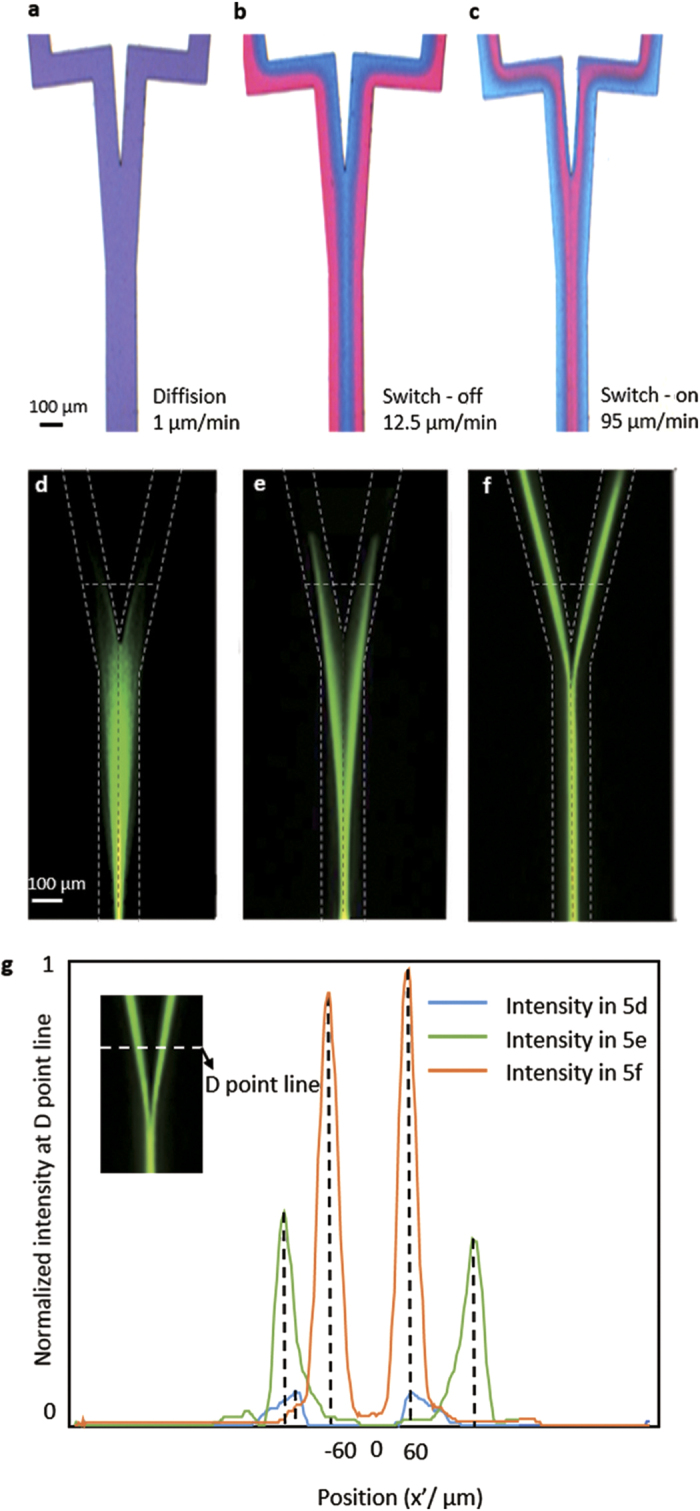
(**a**–**c**) The top view of the flow dyed with pigments in different flow rates, the flow rates are (**a**) *Q*_*1*_ = *Q*_*2*_ = *Q*_*3*_ = *Q*_*4*_ = *1 μl/min*, (**b**) *Q*_*1*_ = *Q*_*2*_ = *Q*_*3*_ = *Q*_*4*_ = *12.5* *μl/min*, (**c**) *Q*_*1*_ = *Q*_*2*_ = *Q*_*3*_ = *Q*_*4*_ = *95 μl/min*. (**d**–**f)** show the light path dyed with rhodamine (6 g) in different flow rates corresponding to the (**a**–**c**). The dotted line denotes the wall of the microchannel and the intensity measurement line of the fluorescent. (**g**) The comparison intensity curve in the “D” point with dependence of on – off state.

To testify the optical switching function of this optofluidic device, we demonstrate the light switch experiments in the Y-junction microchannel. The measured fluorescent images are shown in Fig. 5, the dotted line denotes the wall of the microchannel and the intensity measurement line of the fluorescent. The wavelength of the exciting light is 532 nm (CNI MGL-FN-532/1, 500 mw). Figure 5a–c show the top view of the flow dyed with pigment in different flow rates, the flow rate in 4a is *Q*_*1*_ = *Q*_*2*_ = *Q*_*3*_ = *Q*_*4*_ = *1* *μl/min*, in 5b is *Q*_*1*_ = *Q*_*2*_ = *Q*_*3*_ = *Q*_*4*_ = *12.5* *μl/min*, in 5c is *Q*_*1*_ = *Q*_*2*_ = *Q*_*3*_ = *Q*_*4*_ = *95* *μl/min.* Figure 5d–f show the light path dyed with rhodamine 6 G in different flow rates corresponding to the 5a–c. In Fig. 5a, it is a sufficient diffusion state so the liquid in the microchannel is homogeneous. As a result, light propagated divergence as it shows in Fig. 5d. In Fig. 5b, it is still traditional 2D structures, light will be transmitted to sheath flow and leaked to PDMS before they reach the output fibers. These states are both the switch-off state and light cannot travel through the Y-junction as the RI of PDMS are much larger than two liquid materials. In Fig. 5a,c 3D liquid Y-braches can be realized, light in Fig. 5f can propagate along the channel and smoothly divided into two identical parts without distinct leakage even the RI of liquid is less than PDMS. The state of switch-on and switch-off can be conveniently interchanged by adjusting the flow rates. Figure 5g shows the intensity curve of three on – off state in the “D” point. The purple line corresponds to the Fig. 5d, the intensity of the incident light is border on zero. The blue line corresponds to the Fig. 5e, it is higher than the purple line but obviously lower than the brown line (which corresponds to the Fig. 5f). Although there are some tiny intensity differences (less the four percent) between the two branches in the device, the light intensities of the two branches are considered to be basically the same. So it is easily to distinguish the three on – off state of the device by different intensity in the “D” point.

**Figure 6:**
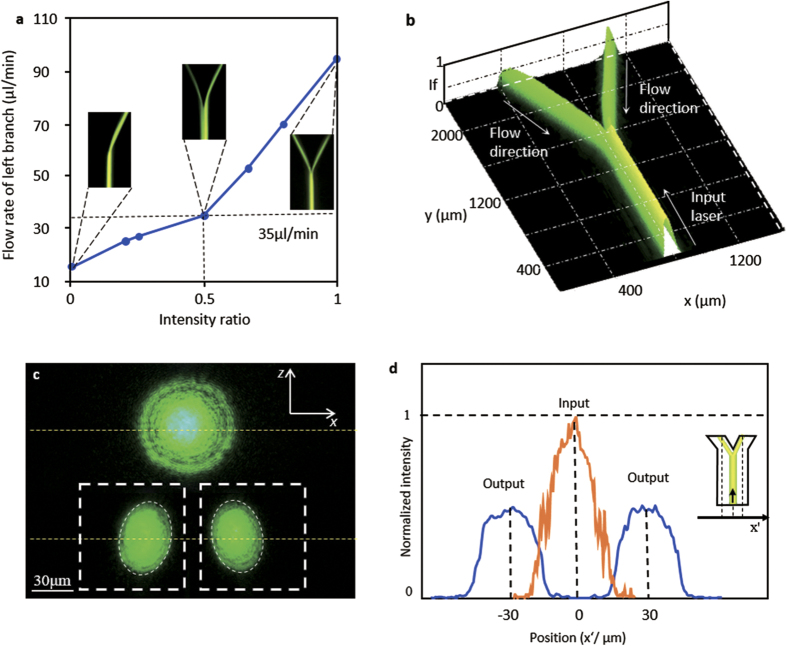
(**a**) The relationship between light ratio and the flow rate of the left EG branch. The flow rate of the right branch is constant. (**b**) The experimental spectral intensity of the optofluidic Y-branch waveguides at switch-on state. The transmission loss through the junction is 0.97 db. (**c**) The light spot from the input fiber and the two output branches. The thick white imaginary line is the wall of the chip and the thin white imaginary is the edge of the light spot. The yellow imaginary lines are the center line of these beam spots. (**d**) The normalization intensity curve of the input light and output light.

The switching properties of the 3D waveguide branches is demonstrated by simulation and realized through experiments. The intensity ratio for two light braches can be controlled by adjusting the relative flow rate of the EG streams, too. Figure 6a displays the correlation between the ratio of intensity for two branches and flow rate of the left curved channel. The flow rate of the EG solution in the left branch is increased from 15 μl/min to 95 μl/min and the other one is maintained at 95 μl/min. It is reasonable to assume that the ratio of intensity for two light branches can be continuously tuned from 0 to 1 by adjusting the flow rates. Figure 6b shows the experimental intensity around the Y-junction in the switch-on state. For uniform concentration of the fluorescent dye, the fluorescence intensity is linear with laser intensity^34^. The intensity of the fluorescence can be used to calculate the relative intensity change of the light accurately. It is all known that the attenuation is defined as 
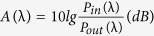
. In which the λ is the operation waveguide of the incident laser, *A(λ)* is the light attenuation through the device, *P*_*in*_*(λ)* and *P*_*out*_*(λ)* are respectively the input light power and output light power. The fraction in this equation means the relative intensity change between the input power and the output power. If we get the fluorescence intensity in the input and output of the device, the transmission loss can be calculated excluding the insertion loss. Thus the intensity in Fig. 6b can be regarded as the practical conditions approximately. An EMCCD (Andor, iXon Ultra) and the inverted fluorescence microscopy (Nikon, ECLIPSE Ti-u) are used to measure the fluorescence intensity in the experiments. The *I*_*f*_ is the normalization intensity of the light in the waveguide. The light intensity in the entrance is 1. So the transmission loss of the 3D optofludic Y-branch is estimated to 0.97 dB when the light ratio is 5:5. When the ratio of the output light is changed, the transmission loss of the device is changed too. The transmission loss is respectively estimated to 0.88 dB when the light ratio is 8:2 and 0.79 dB when the light ratio is 10:0. Compared to its solid brothers, the 3D waveguide branches achieve high flexible while maintain the relative low loss in propagating and splitting, as they are totally independent to solid channels.

The 3D optofluidic branch waveguide can be seemed as a multimode optical waveguide so the modal dispersion must have conspicuous influence on the propagating of the light. Multimode dispersion is the uppermost part among the modal dispersion. It can be described by an approximate formula as^35^ 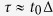, where the 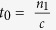 describe the time of the light travels through the per unit length of the medium. *c* is the velocity of light in the vacuum. It is easy to calculate the *τ* by known *n*_*1*_*, ∆* and *c*. Compared to the multimode dispersion, the wavelength dispersion and the material dispersion can be ignored. Because they play a much small role than the multimode dispersion in a multimode optical waveguide when we think the monochromaticity of laser is good and the liquid is isotropy for the light. Figure 6c shows the light spot from the input fiber in the free space and Fig. 6d shows the light spots from the two output branches in the device. A 45-degree microprism (Daheng Optics, GCL-030104A) is placed adjacent to the end of the branches to deflect the light (no dye dissolving solution at this time) so the cross section profile of the beam light can be monitored using an epifluorescence microscope^30^. It is a lateral view through a microprism. The light beam is confined well in the Y-branch waveguide. Compared to the imported beam, the intensity distribution of the output light spot is homogenized through the 3D optofluidic branch waveguide. Figure 6e show the one-dimensional intensity curve of the beam spot. There is slight lateral broadening in the intensity curve of output light. It is a significant characteristic of the multimode waveguide^36^.

## Discussion

The experimental results indicate the three-dimensional optofluidic Y-branch waveguides tuned by Dean flows can achieve both the optical switching function and the tunable ratio of the light. Figure 3a–c show the forming process of the optofluidic Y-branch waveguides influenced by the Dean flows. Its structure is similar to a fiber based Y-branch waveguide but it is totally composed of liquid. The diameter of the core flow can be adjusted by changing the flow rates. Changing the volume ratio between the core flow and cladding flow from 1:4 to 1:1 can adjust the diameter of the core from 20 μm to 50 μm in Fig. 4. Through analysis, it is supposed to perform like a multimode waveguide when the *Δ* = *0.021* and the *λ* = *532 nm*. But if we decrease the Δ and use the input laser with lager λ to make the V can below than the critical value, the 3D optofluidic branch waveguide can also be seemed as a single mode waveguide. Figures 5 and 6a show the 3D optofluidic Y-branch waveguides have good switchable and tunable abilities. Changing the flow rate can change the on – off state and changing the volume ratio of core flow and cladding flow in different branch can change of light ratio of different output. The light will expand to the wall of the channel in traditional 2D optofluidic waveguide in the direction perpendicular to the horizontal plane^37^. But in the 3D optofluidic Y-branch waveguide, the light is basically confined in the core flow. So the device has more excellent confinement of the light than traditional 2D optofluidic chip in Fig. 6d. And the transmission loss is much less than these traditional 2D counterparts, approximately one fifth of the latter^22^. It can have both the high transmission property and the reconfigurability of the optofluidic device.

In conclusion, we demonstrate the switchable 3D optofluidic Y-branch waveguides via Dean flows. The optofluidic Y-branch waveguides consists of two liquid-liquid waveguides and they can make up a 3D liquid braches in the junction of the channel. The numerical simulation and experimental results confirmed the design. Different from the prevailing 2D liquid counterparts, the 3D configuration offer much more freedom in the selection of liquid medium as liquid’s RI can be totally independent to the solid channel structure. This device can direct the light from laser and achieve switch-on or switch-off state by conveniently adjusting the flow rate. The characteristic of the device is similar to a multimode waveguide confirmed by theoretical analysis and experimental results. Further, the relative intensity of two light branches can be tailored from 0 to 1 in similar manner. The transmission loss with the light ratio at 5:5 is estimated to 0.97 db when the operation wavelength is 532 nm. The images of beam spot show the light is confined well in the 3D liquid structures. And the intensity curve shows that the transfer characteristic of the device is accord with a multimode waveguide. It shows that such a Y-branch waveguide may find wider applications in integrated optofluidic communication system.

## Additional Information

**How to cite this article**: Li, L. *et al*. Switchable 3D optofluidic Y-branch waveguides tuned by Dean flows. *Sci. Rep.* **6**, 38338; doi: 10.1038/srep38338 (2016).

**Publisher's note:** Springer Nature remains neutral with regard to jurisdictional claims in published maps and institutional affiliations.

## Figures and Tables

**Table 1:**
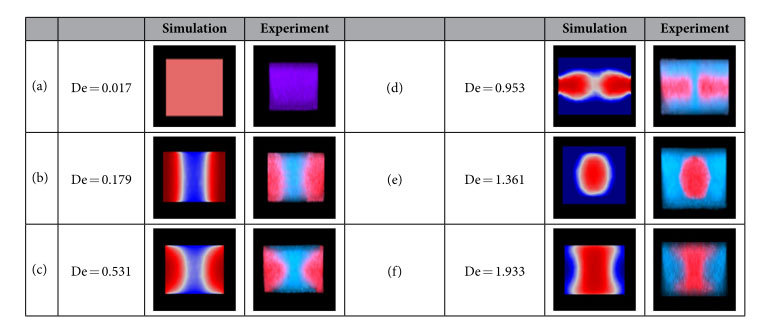
The comparison between simulation and experiment under different Dean numbers.

The flow rates are (**a**) *Q*_*1*_ = *Q*_*2*_ = *Q*_*3*_ = *Q*_*4*_ = *1 μl/min*, (**b**) *Q*_*1*_ = *Q*_*2*_ = *Q*_*3*_ = *Q*_*4*_ = *12.5 μl/min*, (**c**) *Q*_*1*_ = *Q*_*2*_ = *Q*_*3*_ = *Q*_*4*_ = *37 μl/min*, (**d**) *Q*_*1*_ = *Q*_*2*_ = *Q*_*3*_ = *Q*_*4*_ = *63 μl/min*, (**e**) *Q*_*1*_ = *Q*_*2*_ = *Q*_*3*_ = *Q*_*4*_ = *95 μl/min*, (**f**) *Q*_*1*_ = *Q*_*2*_ = *Q*_*3*_ = *Q*_*4*_ = *135 μl/min*. These cross sections are located in the junction of the device. The reconfiguration processes of the 3D optofluidic branch waveguide can be illustrated by the six typical states from low flow rate to high flow rate.
